# Extracts from the Minutes of Atlanta Medical Society

**Published:** 1866-05

**Authors:** 


*Extracts from the Minutes of Atlanta Medical Society.*

ATLANTA, March, 1866.

Report of Cases being in order—

Dr. O'Keefe reported a case with the following history: S. W., aged about forty years, light hair and complexion—by occupation a merchant—had spent several years of his life in the British West Indies, and suffered from the endemic diseases of that country, which resulted (he was so informed by his physicians) in enlargement of the liver and spleen, and for which he had been several times ptyalized. He was also informed by his medical attendants, that he had some form of cardiac disease.

Nothwithstanding this formidable complication of maladies, he enjoyed moderately good health for several years past, whilst a resident of this city, until the past winter, when, whilst traveling on business, he was seized with acute dysentery. He returned to this city in December last, at which time he placed himself under Dr. O'K.'s treatment.

He was, at this time, suffering from sub-acute—perhaps it might be called chronic—dysentery, from which he gradually recovered. On the eighth February last, being still somewhat feeble, but entirely relieved from the dysenteric condition, he began to experience dimness of vision to such an extent that he could neither read writing nor print, accompanied by a dull aching pain in the eye-balls, over the brows, and somewhat over the whole scalp, as well as within the cranium. Deep-seated inflammation of the eye being apprehended, he was cupped freely over both temples, an anodyne application kept over the eyes and brows, and one scruple of calomel administered. The calomel acted very freely on the bowels, (perhaps half a dozen times,) so that it was not deemed necessary to follow it with any other purgative. The eye and head symptoms were much benefitted by these means—indeed, they all disappeared except the cloudy vision, which was improved. In a few days after taking the mercurial, a very troublesome ptyalism was developed, which required attention for two weeks, and which left his system in a feeble and irritable condition.

About the time he was fairly over his salivation, certain embarrassments of respiration began to show themselves; he found his "breath shorter"—that he slept with his head and shoulders more elevated than ususal, and that, sometimes in the night, he was compelled to jump up suddenly in bed and assume the erect posture. At this time I was visiting him once or twice a week, and was sent for to remedy these new developments. I found him in bed with his shoulders propped up and his respiration impeded. He had a troublesome cough which he attributed to a recent cold. The chest was carefully explored in the sitting posture, with the following result: Left side dull on percussion, nearly as high as the nipple, in front, with little or no respiration in the same region. A cardiac murmur was distinctly heard on a line with the apex of the heart, about two inches to the inner side of the usual point of impulse; i. e., very near the point of the ensiform cartilage, and the impulse of the apex was more distinct at this point than at the normal situation. This murmur accompanied the first sound of the heart, and became more distinct as it was traced from base to apex, and less so when followed in the opposite direction. From these circumstances, in regard to the murmur, I infer that the mitral valve was its seat.

Posteriorly there was some dullness on percussion, but not sufficiently marked to be of serious import. Respiration was rather feeble, but was heard at the base of lung, especially close to the vertebral column.

*Right side.* The usual hepatic dullness extended to the seventh rib anteriorly, and a little beyond; posteriorly the percussion resonance was dull, almost as high as the inferior angle of the scapula, and but little respiration over the same region; large crepitant rale, the *crepitans redue* of the stage of resolution of acute pneumonia existed, to a limited extent, in this region.

In percussing the abdomen, intestinal resonance was absent over a belt of that cavity two to three inches in width, below the edge of the ribs, and extending from the right to the left hypochondrium. Indeed, on the right side, the dullness extended almost as low down as the iliac fossa. The abdomen, too, was somewhat tense and distended, but no fluctuation could be discovered.

The symptoms and signs in the case at this point of its history, were interpreted as follows: The dullness on the left side of thorax anteriorly, was regarded as dependent on hypertrophy of the heart, especially of its left side—a result of frequent occurrence in consumption, with disease of the mitral valve. The dullness and crepitation on the right side, posteriorly, were due (it was thought) to latent pneumonia. The dullness in the epigastric and hypochondriac regions, were deemed to mean enlargement of the liver and spleen of long duration. Thus, in brief, the diagnosis made of his case was, hypertrophy of the heart, due to organic disease of the left auriculo-ventricular valve; latent pneumonia of right lung, posteriorly; chronic enlargement of liver and spleen. (NOTE.—I should have stated, in giving his history, that for several years of his life, he had been "a free drinker.")

I visited him again in a few days, and found all his symptoms aggravated, especially the dyspnoa; for one or two nights he could not lie in bed, but spent them sitting in a chair.

His chest was examined again in the sitting posture, and a uniform and regular dullness was found encircling the thorax as high as the nipples, and extending downwards to its base, and respiration was absent over the same space, anteriorly, posteriorly and latterly. The crepitant rale on the right side, posteriorly, was gone, and tubal respiration had taken its place, which was especially distinct at the inferior angle of the scapula, and round towards the axillæ. It was now evident that there was effusion in both pleural cavities, and that hydro-thorax was added to his other difficulties. He was put upon a tonic alterative and diuretic course of treatment, and a brisk cathartic of jalap and cream tartar administered. Four days from this time I visited him again; the cathartic had purged him very freely—indeed, was still purging him, without any consequent prostration; the dyspnoa was greatly relieved; he slept quite well and required but little elevation of the shoulders; his ortho-pnoa was gone. The dullness had disappeared, in a great measure, from the left side of chest, and respiration had returned, but there was no change in the physical signs of the right side, nor of the abdomen. What cleared up the left thorax? What removed the dullness and restored the respiration? If it was dropsy, as supposed, and the purging removed the greater part of it on the left side, why was not the right side equally benefited? Supposing the physical signs of both sides of the chest to be due exclusively to effusion within their cavities, would it be possible for purgation to remove it from one side and not the other?

---

NOTE.—The condition of this man on this, April 15th, has changed but little since the last record made of it in the report. The left chest is clear, the right still dull, but gradually clearing up. I believe the dullness and absence of respiration due to condensation of lower lobe of right lung—perhaps chronic hepatization. The feet and legs are œdematous, the abdomen enlarged, but no fluctuation perceptible. Indeed, the areola tissue, generally, is somewhat distended with effusion. Dyspnoa still troublesome, and yet the evidence of dropsy of the chest has well nigh disappeared. May it not be due to œdema of the areola tissue of the lungs? On the whole the prognosis is unfavorable.

Dr. J. M. Boring mentioned a case of small-pox coming under his notice and management, having anomalous symptoms. The eruption, though sufficiently marked to render the diagnosis undoubted, was by no means so general as usual. Very malignant symptoms were present from the first visit. The skin assumed an ecchymosed appearance, with occasional blebs, filled with sanious fluid, on various parts of the body.

Dr. D. C. O'Keefe had seen a similar case to some extent, and so unlike the usual appearance of ordinary variolous eruption was it, that, at a consultation had to determine the diagnosis, it was decided not to be variola. It being in the country, amid numerous unprotected persons, the results proved the incorrectness of the decision of the consulting physicians.

Both cases proved fatal in a few days.

Dr. R. C. Word reported a case of ulceration of the os uteri, in which he adopted the novel mode of cauterizing the ulcers, by sponge saturated with solution of nitrate of silver being placed in front of the piston of a female syringe. The instrument then introduced into the vagina, with the perforated extremity coming in contact with the os, the piston is forced against the sponge, and the solution pressed out against the diseased organ. He allows the patient to apply the remedy herself, and, in the case referred to, with the best results.

Dr. O'Keefe thought the uncertainty of the exact position of the cervix and os, particularly in a diseased uterus, would render the application altogether unreliable.

---

Atlanta, March 3, 1866.

Reports of cases being in order—

Dr. J. F. Alexander reported a case of purpura, in which quinine was successfully used. He was led to its use by the periodical increase of the prominent symptoms of the disease in this case. Regular daily exacerbations were observed, and, as was his custom in other diseases assuming this periodical character, quinine was given. This report led to a discussion of the general effects and modus operandi of quinine.

Dr. Word thought it particularly applicable in the various forms of congestion; hence, in incipient pneumonia, where local congestion of a portion of the lung exists, and in intermittent fever, where the internal organs are engorged at the expense of the peripheral circulation, it is found to correct the morbid condition. He had also witnessed its salutary effects in menorrhagia; and, in a case where the remedy was used for some purpose, during the healthy catamenial flow, the discharge was arrested, as it would seem, from the effects of the quinine.

Dr. Alexander had derived benefit to his patient from the use of quinine in suppressed menstruation.

Dr. J. G. Westmoreland thought that these seemingly contradictory statements, or antagonistic actions of the remedy, may be *harmonized* by a consideration of its direct action upon the system. By keeping in mind the facts that quinine acts primarily upon the medulla spinalis, and gives tone and energy to that nervous centre when inactive from malarious poison or other causes, the difficulty is easy of solution. The various internal organs, and the capillary and general circulation—being dependent upon the influence of the spinal nerves for the performance of their functions—may suffer the many, and, under varied circumstances, even opposite forms of disturbance, by inactivity of the cord. In a particular condition of the uterus, depressed nervous influence may lead to excessive discharge, while under other circumstances the normal flow may be suspended from the same state of the nervous system. Hence, quinine, by giving energy to the nervous centre, indirectly relieves these opposite forms of functional derangement dependent on enervation.

---

Atlanta, March 10, 1866.

Dr. D. H. Connally reported a case of threatened miscarriage at five months, in which there was some dilatation of the os, with slight hemorrhage. The pains, which at first observed short intervals, were measurably controlled by opium.

He desired the opinion of members in regard to the probability of arresting the pains permanently under the circumstances, (the case being at the time in progress.)

Several members thought, from the symptoms narrated, that there was no cause to despair of permanently arresting the contractions of the uterus without miscarriage.

Dr. W. F. Westmoreland alluded to the importance of ascertaining whether or not the child be living. He had seen females subjected to unnecessary inconvenience, and perhaps danger, by quieting the action of the womb, and keeping the patient in bed several days under strict directions and regular medications, to prevent the expulsion of the fœtus, which, it was afterwards ascertained, had been dead the whole time.

Dr. Redwine had witnessed the difficulties arising from the failure to detect the death of the fœtus. A case under his care, in which abortion was threatened at the third or fourth month, went on to the full period of utero gestation, and the woman was then delivered of a dead fœtus, with development corresponding with the time of the threatened abortion, proving to his satisfaction. that the dead embryo had remained in the uterus for five or six months.

He agreed with other members in the opinion that the appearance of slight hemorrhage and the commencement of dilatation of the os uteri, were not sufficient reasons to despair of arresting the expulsive tendency, and preserving the life of the fœtus. He had succeeded where considerable hemorrhage was present.

Dr. Word reported the case of a child fourteen months old, afflicted with pneumonia, in which veratrum failed to produce the usual sedative action upon the heart. It was given to the extent of producing nausea and vomiting, without materially lessening the frequency of pulsations. He desired to get the experience of other members in the use of this remedy with children.

Dr. Redwine, and other members had seen the remedy administered to adults with the same results, but none had noticed the age having anything to do with this failure to produce sedation.

Dr. J. G. Westmoreland reported a case of acute articular rheumatism, in a female child of eight or nine years old, which promptly disappeared under the action of a blister over the spine and the internal use of full doses of quinine. He mentioned this case in order to call the attention of the Society to this plan of treating rheumatism. Did not consider quinine salutary, or even beneficial, in many cases of chronic articular and nervous rheumatism. While this is the case, the disease in the acute stage, affecting the joints, is as thoroughly subject to the influence of quinine as malarial fever.

The exact plan of treatment pursued, was as follows: About the 15th March, 1866, called to see the little girl alluded to; found the knees, ankles and right elbow swollen, tender, and most of the time—the right knee particularly—quite painful. The febrile symptoms were high, with an inability to move or be turned in bed without greatly increasing the suffering. These symptoms, commencing two days previously, had gradually grown worse up to the time of the visit. Prescribed three grs. quinine, with ten drops tr. opium, every three hours till three doses are taken. Also applied a blister nearly the whole length of the spine, one inch and a half wide. Being several miles from town, the father was requested to report the condition of the patient next day, which he did, with the information that much relief was afforded, but the symptoms, to some extent, still remained. Ten or twelve grs. more of quinine were prescribed, in three doses, with the same amount of opium, and observing the same intervals as before. Requested further report from the case in a day or two, if the disease gave any further trouble. Nothing more was heard from the case until she was walking about the yard several days afterward.

Dr. Henry Orme had been in the habit of relying upon quinine in the treatment of this disease, and was satisfied with its effects.

